# High-Quality In Vivo Chemical Probes for Protein Kinases Disclosed in 2024

**DOI:** 10.1021/acsptsci.5c00293

**Published:** 2025-07-17

**Authors:** Ricardo A. M. Serafim, Matthias Gehringer

**Affiliations:** † Department of Organic and Pharmaceutical Chemistry, School of Engineering, Institut Químic de Sarrià (IQS), 82824 Universitat Ramon Llull (URL), Vía Augusta 390, 08017 Barcelona, Spain; ‡ Department for Medicinal Chemistry, Institute for Biomedical Engineering, Faculty of Medicine, 9188 Eberhard Karls University Tübingen, Auf der Morgenstelle 8, 72076 Tübingen, Germany; § Cluster of Excellence iFIT (EXC 2180) ‘Image-Guided & Functionally Instructed Tumor Therapies,’ University of Tübingen, 72076 Tübingen, Germany; ∥ Department of Pharmaceutical/Medicinal Chemistry, Institute of Pharmaceutical Sciences, Eberhard Karls University Tübingen, Auf der Morgenstelle 8, 72076 Tübingen, Germany

**Keywords:** chemical probes, protein kinase inhibitors, target validation, drug discovery, understudied kinome

## Abstract

Protein kinases are highly relevant drug targets, yet a significant fraction of the human kinome remains underexplored. Highly potent and selective small-molecule inhibitors used as chemical probes are invaluable tools for enabling the validation and translation of new kinase targets. This review provides an overview and analysis of the high-quality in vivo chemical probes for protein kinases published and deposited at the Chemical Probes Portal in the year 2024. We discuss the design strategies, molecular mechanism of action, details of their use in vitro and in vivo, as well as their application in target (in)­validation. We also highlight the importance of wisely selecting chemical probes and encourage best practices in using such tool compounds.

## Graphical abstract

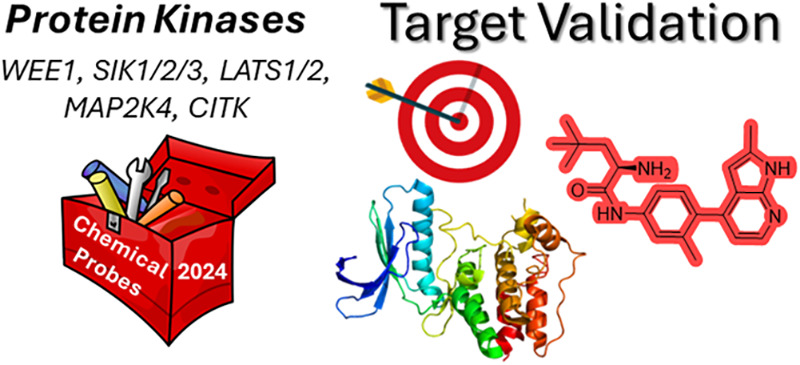

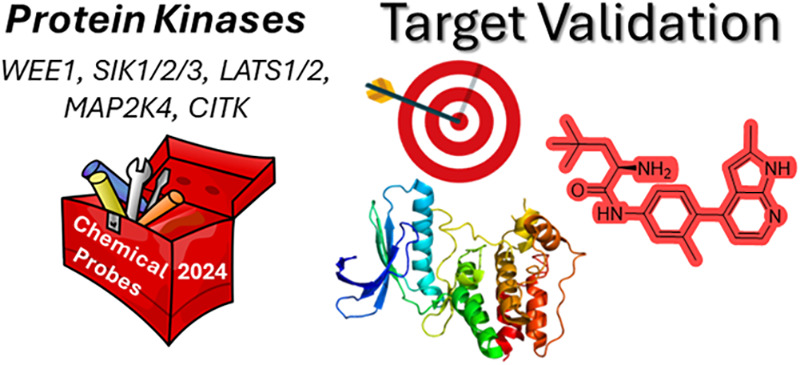

Drug discovery and development are time-consuming and costly processes. From early discovery phases to FDA approval, a new drug often takes between 10 and 15 years at an estimated cost reaching from ∼700 million up to 5 billion dollars, a value that is growing substantially over time.^1–3^ The high expectation for an increase in the number of drug approvals in the postgenomic era fueled by the availability of sequencing data and the emergence of new technologies has been contrasted by a decline in approval rates, especially in the first decades of this century.^4,5^ Despite the remarkable recent ten-year rolling average of 46 drugs approved per year,^4^ the attrition rate in the clinical phase 2 achieved the substantial value of ∼75% between the years 2005 and 2009.^5^ An in-depth study found that among many possibilities (safety, commercial, strategic, pharmacokinetic), lack of efficacy was the main reason for the failure in advanced clinical stages (i.e., phases 2 and 3) between the years 2007 and 2015.^6–8^ These outcomes are largely a consequence of poor target selection, frequently stemming from inadequate preclinical target validation, which leads to an insufficient understanding of the molecular mechanisms underlying the disease.^5^

Rigorous target validation in the early preclinical stages is a fundamental practice in drug discovery to mitigate attrition rates in clinical trials. The main goal is to confirm or refute the direct correlation between target modulation and the observed disease phenotype, which is crucial for the selection of the most suitable molecular target to interfere with the desired pathological process.^9,10^

Protein kinases are among the most important human protein families involved in intracellular signal transduction. These enzymes catalyze the transfer of a phosphate group from their cosubstrate ATP to a protein substrate. The human genome encodes more than 500 protein kinases, collectively referred to as the “kinome,” which plays a direct or indirect role in essentially all cellular signaling pathways.^11^ Since the early 2000s, when the first deliberately developed kinase inhibitor imatinib entered the market,^12,13^ the enormous potential of protein kinases as drug targets has been confirmed by a substantial number of drug approvals in the last 25 years. At the time of writing, 85 small-molecule protein kinase inhibitors were approved by the US Food and Drug Administration (FDA),^14^ primarily for the treatment of different types of cancers but also for inflammatory and immunological disorders.^15^ However, there is still a significant fraction of the kinome which has not yet been explored.^16^ The Illuminating the Druggable Genome (IDH) program run by the US National Institute of Health (NIH) showed that ∼30% of the human kinome is comprised of protein kinases with unknown or poorly understood biological functions (sometimes referred to as “dark kinases”).^17^ Along the same lines, recent analyses from the Bajorath group^18,19^ showed that besides nearly half of the protein kinases still lacking narrow spectrum inhibitors, the current protein kinase inhibitor drugs cover only ∼25% of the human kinome, leaving opportunities for medicinal chemistry and drug discovery in the kinase field.

The initial study of protein function is usually conducted by genetic approaches, such as RNAi^20^ and CRIPSR-Cas9 ^21^ systems. However, in order to start a robust translational drug discovery program, complementary evidence by chemical approaches is essential to ensure the druggability and a robust link between pharmacological target modulation and disease.^22,23^ In this context, the so-called “chemical probes,” i.e., highly specific small molecules that act as pharmacological modulators, are extremely powerful tools for elucidating the biological function and druggability of unexplored proteins.^24,25^ Such small molecules allow researchers to answer mechanistic questions in a relevant biological system and establish possible correlations between specific chemical target modulation and the resulting phenotype.^26–29^ Therefore, development of chemical probes for kinases is of great importance for the mechanistic and therapeutic exploration of the understudied part of the kinome.^30,31^ In order to be classified as a high-quality chemical probe for protein kinases according to the criteria of the Chemical Probes Portal^32^ and the Structural Genomics Consortium (SGC), a small-molecule inhibitor must meet the following criteria: biochemical potency <100 nM (at an ATP concentration equaling its *K*_m_ value); robust proof of cellular target engagement <1 μM, e.g., by biophysical methods or biomarkers; and selectivity over 30-fold versus a broad panel of kinases with exception of closely related paralogues.^33^ Additionally, a structurally closely related negative control should be available with >100-fold weaker potency on the kinase target,^33,34^ a criterion that is unfortunately often not fulfilled. Besides classical noncovalent inhibitors, covalent protein kinase inhibitors and kinase degraders (for example, PROTACs – proteolysis-targeting chimeras) can be used as chemical probes.^31,35–37^ Additional criteria have been defined for such modalities and can be found elsewhere.^38–40^

The Chemical Probes Portal is one of the most important open science resources for the global biomedical research community that compiles, evaluates, and promotes free access to chemical probes. It serves as a valuable repository of easily accessible information about chemical probes to aid scientists, reviewers, and editors in their experiments and deliberations.^41,42^ In addition to providing a star rating system describing the suitability of chemical probes for cell and in vivo studies, the Chemical Probes Portal now also features a collection of “unsuitables,” i.e., compounds that are frequently (mis)­used as probes in the current literature but are not suitable for this purpose. Together, these resources help to promote mindful use of chemical probes in biomedical research and counteract the continued use of poor historical tool compounds despite the availability of better alternatives. It is also worth noting other portals and resources related to chemical probes,^43^ including the Chemical Probes web page of the Structural Genomics Consortium,^44^ the OpnMe portal from Boehringer Ingelheim,^45,46^ and Probe Miner from the Institute of Cancer Research (ICR).^47,48^

Herein, we present a compilation and a critical analysis of the most recent high-quality (nondegrader) chemical probes for protein kinases deposited at the Chemical Probes Portal and with the corresponding papers published throughout the year 2024. Sections are arranged per kinase target, and only compounds that meet the aforementioned criteria and have been rated with a minimum of 3 out of 4 stars in cellulo and in vivo by the expert reviewers of the Chemical Probes Portal have been included. We discuss and showcase their chemical development, biological and PK/PD properties, and application in target (in)­validation.

## WEE1

Wee1 is an atypical nuclear tyrosine kinase that plays an important role in cell division. Through the phosphorylation of CDK1/2-cyclin substrates, Wee1 regulates the G2/M transition and S phase checkpoint, avoiding premature cell mitosis.^49,50^ Wee1 inhibition can be strategically used to target some cancer cells by forcing these cells to an earlier entry to mitosis without giving the DNA repair machinery the time to properly start working, which leads to cellular death or senescence.^51–54^ Furthermore, Wee1 overexpression has been related to poor prognosis in many kinds of cancers.^55–57^ The pyrazolopyrimidinone AZD1775 (adavosertib, **1**)^58,59^ (Figure 1) from AstraZeneca was the pioneering Wee1 inhibitor to enter phase II clinical trials, followed by the structurally related Zn-c3 (azenosertib, **2**)^60^ (Figure 1), with the latter receiving orphan drug designation for the treatment of osteosarcoma and rare pediatric diseases in 2021 and currently being evaluated in clinical phase II as a single-agent treatment for solid tumors as well as recurrent or persistent uterine serous carcinoma.^60^

**Figure 1:**
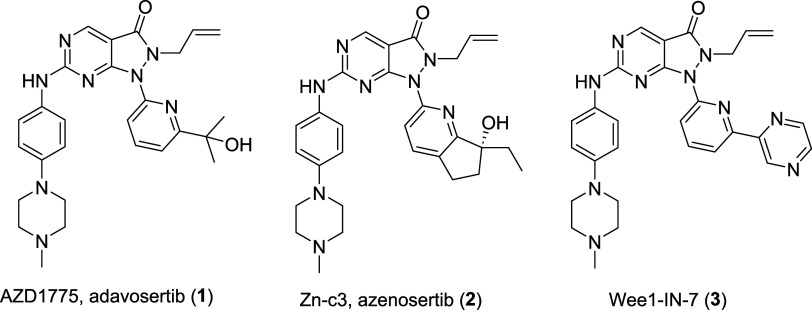
Molecular Structure of the Wee1 inhibitors clinical candidates **1**, **2**, and chemical probe **3**.

However, compound **1** has been facing issues with significant clinical side effects, likely related to its poor overall kinome selectivity (see the discussion below).^60–62^ Therefore, Wang and co-workers^63^ aimed at developing more selective and potent Wee1 inhibitors based on the structure of compound **1**.

Structural analysis of the Wee1 and related kinases’ ATP-binding sites, together with molecular docking experiments, showed the potential to explore new interactions at the Wee1 P-loop region, especially π–π stacking with the phenyl ring of Phe310. After a SAR exploration replacing the *tert*-butyl alcohol tail of compound **1** (PDB code: 5V5Y) with different aromatic rings, compound **3** (Wee1-IN-7, Figure 1) containing a pyrazine group was obtained. This compound demonstrated good biochemical inhibitory and cellular antiproliferative activity (Wee1 IC_50_ = 2.1 nM and A427 IC_50_ = 84 nM, respectively) and favorable pharmacokinetics in mice.^63^ Despite further molecular modifications in other parts of the scaffold, none of them was able to outperform the PK/PD properties of compound **3**. After oral administration, compound **3** showed an improved level of tumor growth inhibition (88%) when compared to its prototype **1** (70%) and the other pyrazolopyrimidinone-based clinical candidate **2** (80%) in a mouse xenograft model. Flow cytometric assays confirmed the antitumoral effect of compound **3** by inducing apoptosis as well as a dose-dependent interference in the cell cycle progression in line with Wee1 inhibition.^63^

A cross-screening against an enzyme assay panel including 225 kinases (ICE Bioscience Inc.) at 1 μM confirmed the narrow selectivity profile of compound **3**, showing full inhibition of Wee1 and only two off-target kinases (GAK and MAP3K19) with more than 90% of inhibition.^63^ Although this screening revealed a promising selective profile of inhibitor **3**, it would be helpful to test this compound in a panel that covers an even broader fraction of the kinome and evaluate the IC_50_ values for the most relevant off-targets. Notably, the above-mentioned clinical candidates (**1** and **2**) have been evaluated in a larger panel with approximately 475 kinases (Thermo Fisher).^60,64^ Here, compound **1** showed inferior selectivity, inhibiting 13 kinases ≥ 90% at 1 μM, and compound **2**, at the same concentration, hit only 5 kinases ≥ 90% (Wee1, PLK2, YSK4, EGFR d747–749/A750P, and PLK3), which may indicate a similar level of selectivity as observed for **3**.^60^ Nevertheless, in a side-by-side acute toxicity experiment in mice, no significant decline in the body weight of the animals was observed even after 14 days of treatment with compound **3**. In contrast, the animals exhibited severe weight loss and did not survive after only 7 days of treatment with compound **1**, suggesting a superior safety profile of inhibitor **3** even at the maximum concentration tested (2000 mg/kg).^63^ Its relatively clean selectivity profile suggests compound **3** as the most suitable chemical tool to study Wee1 kinase, especially when considering in vivo applications. However, it lacks a negative control compound, and ideally, a second inhibitor with a complementary chemotype should be used for validation, a guideline that should always be considered.

## SIK1, SIK2, and SIK3

The salt-inducible kinases (SIK1–3) are serine/threonine kinases from the adenosine monophosphate–activated kinase (AMPK) subfamily of the calcium/calmodulin-dependent kinase (CaMK) group.^65^ SIK1–3 have pivotal roles in the regulation of homeostasis and metabolic stress.^66,67^ In addition, studies have shown the importance of SIKs in the modulation of the activity of the innate immune system^68–70^ (mainly SIK2/3), blood pressure regulation^71,72^ (mainly SIK1), osteoporosis,^73^ and tumorigenesis.^68,72,74^ Several nonselective pan-SIK inhibitors from different chemotypes have been described in the past, such as dasatinib (**4**), bosutinib (**5**),^70^ HG-9–91–01 (**6**),^68^ YKL-05–099 (**7**),^75^ GLPG3312 (**8**),^76^ and ARN-3236 (**9**)^69^ (Figure 2). In 2021, efforts from the Knapp group generated the pan-SIK inhibitor **10** (MRIA9, Figure 2), which has a slightly better potency toward SIK2/3, good cellular target engagement, and excellent selectivity in a panel of 443 kinases (Reaction Biology Corp.) at 1 μM using the radiometric activity assay format, showing only PAK2/3 as off-targets.^77^ Another compound, GLPG4399 from Galapagos NV, has entered clinical trials for the treatment of inflammatory arthritis as a first-in-class SIK3 inhibitor, but the structure and additional information have not yet been disclosed.^78^

**Figure 2:**
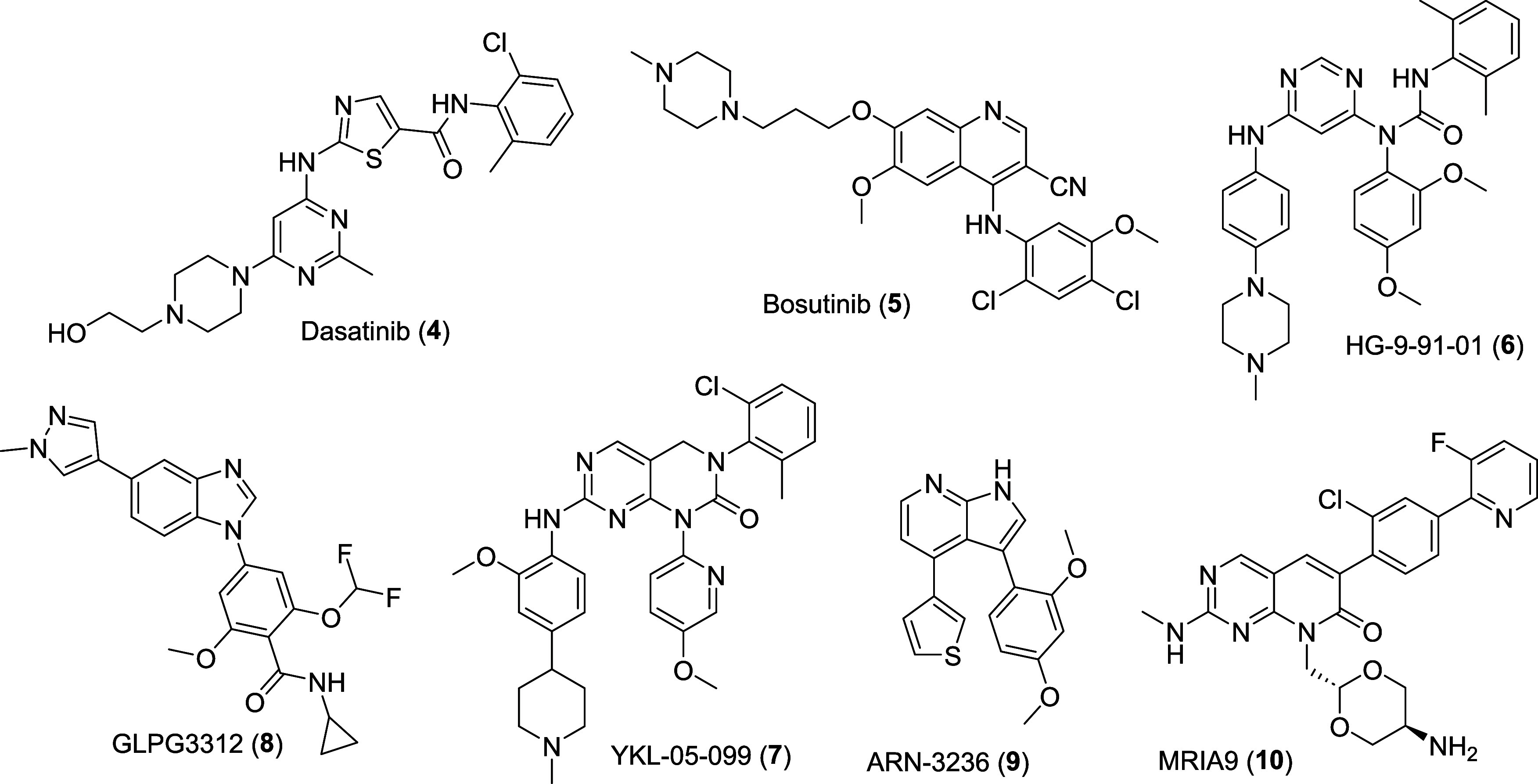
Molecular structure of pan-SIK inhibitors.

Due to the need for isoform-selective SIK chemical probes and possibly drug candidates, Babbe and co-workers^79^ identified compound **11** (Figure 3A) as an attractive hit through an HTS campaign using the Janssen chemical library. The compound shows 18-fold selectivity for SIK2 over SIK3 (*K*_i_ = 0.022 and *K*_i_ = 0.406 μM, respectively, in an ADP-Glo assay). A modeled SIK2-**11** complex showed the phenyl group being oriented toward the back pocket, which suggested that small substituents could more favorably fit with the smaller side-chain of the threonine gatekeeper residue than the larger methionine or leucine present in the other AMPK members (see ref 79, Supporting Information for details). Moreover, positively charged substituents interacting with the unique Glu103 at the SIKs catalytic site were postulated to provide additional selectivity against the homologous members in the SRC family, which have cysteine at the same position. The structure-guided molecular optimization successfully yielded the pyrrolidine derivative **12** and the azetidine derivative **13** (SIK2 *K*_i_ = 1.15 nM and 2.20 nM, respectively – Figure 3A), with the latter achieving 100-fold selectivity over the SRC kinase LCK, improved microsomal stability, and in vivo pharmacokinetics. In addition, an X-ray structure of **12** (Figure 3D) bound to a MARK2-SIK2 chimera (PDB code: 8TXY) confirmed the predicted interactions of the nitrile at the gatekeeper as well as the pyrrolidine making ionic contact with the Glu103 residue. Compounds **12** and **13** have an isoform selectivity of 30-fold toward SIK1/2 over SIK3. In the cross-screening against 327 kinases (Eurofins), using a radiometric kinase activity assay, for both compounds, only 4 off-targets were found (ABL, LCK, RIPK2, and YES) with >50% inhibition at 1 μM concentration, and all of them also share a threonine gatekeeper residue.^79^ This excellent selectivity degree of **12** and **13** outperforms the previous selective SIK inhibitor **10**, which has 8 off-targets in a ratio of <100-fold versus SIK2.^77^ In vitro and in vivo experiments demonstrated that **13** was able to reduce levels of proinflammatory cytokines while inducing anti-inflammatory interleukin-10. Oral administration suppressed wasting disease and colitis in mouse models, suggesting that selective SIK1/2 inhibition can mitigate colonic inflammation driven by activated immune cells without toxic effects.^79^

**Figure 3:**
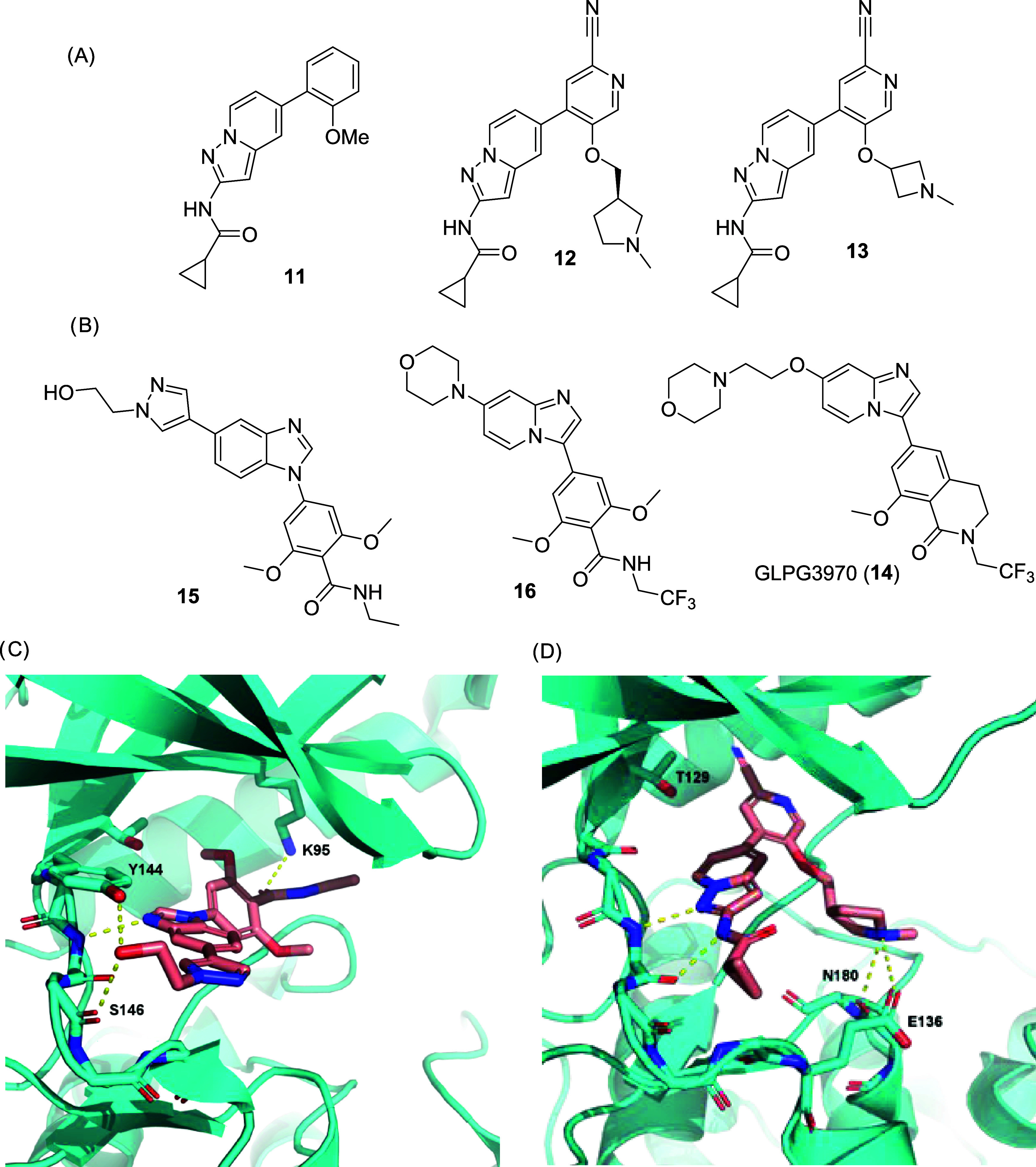
Molecular structure of SIK inhibitors: (A) Hit SIK1/2 inhibitor **11**, SIK1/2 isoform-inhibitor **12**, and SIK1/2 chemical probe **13**. (B) Hit SIK2/3 inhibitor **15**, optimized SIK2/3 inhibitor **16**, and SIK2/3 chemical probe GLPG3970 (**14**). (C) X-ray crystal structure of **15** bound to SIK3 (PDB code: 8OKU). (D) X-ray crystal structure of **13** bound to a MARK2-SIK2 chimera (PDB code: 8TXY). Hydrogen bonds are depicted as yellow dashed lines. Side chains of relevant residues are depicted as sticks and labeled.

In 2024, Peixoto and co-workers^80^ described the development of the selective dual SIK2/3 inhibitor GLPG3970 (**14**, Figure 3B). After a previous hit discovery, elucidation of the binding mode of **15** (Figure 3B,C) complexed with SIK3 (PDB code: 8OKU),^76^ and analysis of the alignment with SIK1/2, the authors hypothesized that exploration on the different residues around pyrazole (Tyr98 and Tyr144 for SIK2/3, respectively, and Phe105 for SIK1) could be suitable to achieve isoform selectivity.^80^ Initially, the replacement to the nonaromatic morpholine followed by a scaffold hopping to an imidazo­[1,2-*a*]­pyridine core gave compound **16** (Figure 3B) with a slightly higher SIK2/3 selectivity over SIK1. After an extensive SAR evaluation, introduction of a linker between the imidazo­[1,2-*a*]­pyridine and morpholine in combination with a conformational restriction by lactam generation while maintaining the *N*-trifluoroethyl group yielded compound **14** with potent SIK2 and 3 inhibition (IC_50_ = 7.8 and 3.8 nM, respectively), more than 30-fold selectivity against SIK1 (IC_50_ = 282 nM), and negative results in a CYP time-dependent inhibition assay. Beyond SIKs, compound **14** only inhibited three other proteins with more than 50% inhibition (RIPK2 = 79%; ABL1 = 58% and MKNK2 = 54%) at 1 μM concentration in a panel of 372 kinases (Eurofins). However, only RIPK2 (IC_50_ = 78.4 nM) was confirmed as a potent off-target by following IC_50_ determination (ABL1 and MKNK2 IC_50_ = 1,095 nM and 1,074 nM, respectively).^80^ It is worth mentioning that RIPK2 is also the main off-target of pan-SIK inhibitor **8**, mentioned above.^76^ In vivo PK evaluation showed that compound **14** had promising oral bioavailability in rats and dogs (55.9 and 41%, respectively) and moderate total clearance and low unbound plasma clearance. Cellular pharmacologic studies demonstrated that compound **14** had immunomodulatory effects via TNF-α inhibition and stimulation of IL-10, with similar results obtained in whole blood from healthy volunteers. Inhibitor **14** was also very efficient in reducing chemically induced colitis in mouse models in a dose-dependent manner.^80^ Clinical results have not yet been disclosed.

In summary, compounds **13** ^79^ (3-star rating in vitro/in vivo) and **14** ^80^ (4-star rating in vitro/in vivo) are two recent high-quality chemical probes for SIKs. When used at the recommended concentration, those inhibitors can be excellent tools for in vitro and in vivo investigation of the biology and function of different SIK isoforms (**13** for SIK1/2 and **14** for SIK2/3 – yet both without negative controls) and, perhaps in the future, also in the clinics for the treatment of inflammatory diseases.

## LATS1 and LATS2

The YAP/Hippo pathway was initially discovered in *Drosophila melanogaster* and is highly conserved in humans, playing pivotal role in numerous biological processes linked to tissue regeneration.^81^ YAP activity is regulated by the closely related LATS kinases (LATS1 and LATS2), which inactivate and label YAP for degradation through direct phosphorylation.^82–84^ Because of their negative regulatory effect, LATS kinases are promising molecular targets for the induction of YAP signaling.

As reported in 2021, Kastan and co-workers^85^ identified the YAP activator **17** (Figure 4) in a phenotypic HTS, which was then identified to possess LATS inhibitory activity. Despite the promising activity in biochemical and cellular assays (LATS1/2 IC_50_ = 0.2 nM and EC_50_ = 510 nM for inhibition of YAP phosphorylation in HEK293A cells), evaluation in a broader panel of 314 kinases (panel type not mentioned) showed inhibitor **17** to possess a low degree of selectivity hitting 34 off-targets stronger than the primary target at 1 μM concentration. One year later, another phenotypic screen reported by Aihara and colleagues^86^ identified the acylhydrazones **18** and **19** (Figure 4) as LATS inhibitors (**19:** IC_50_ = 4.1 nM and 3.9 nM for LATS1 and LATS2, respectively). Again, a poor degree of selectivity was found when **19** was screened against 321 kinases (Carna Bioscience), inhibiting 16 other AGC kinase family members by more than 65% at 100 nM concentration (such as MSK1, p70S6K, PKACα/β, and SGK2/3). The lack of selectivity of those compounds could directly influence the interpretation of the observed phenotypes in mechanistic studies, reinforcing the importance of the development of high-quality chemical tools for studying LATS biology in an appropriate manner.

**Figure 4:**
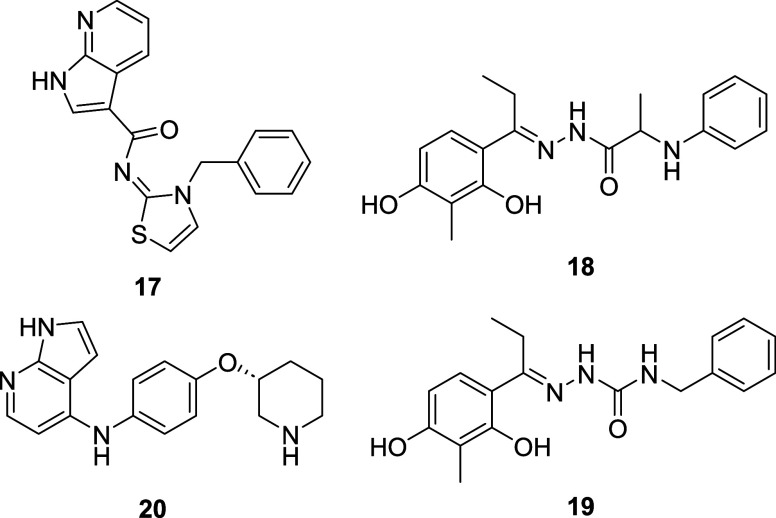
Molecular structure of the nonselective LATS inhibitors **17**, **18**, **19**, and chemical probe **20**.

A team of researchers from Novartis led by Namoto and Tchorz^87^ recently described the discovery of the highly potent and dual selective LATS1/2 inhibitor **20** (Figure 4). After a screening campaign followed by extensive cycles of medicinal chemistry optimization, compound **20** was obtained as an inhibitor with nanomolar potency against LATS1 (IC_50_ = 1.41 nM), showing strong suppression of YAP phosphorylation in JHH5 cells (IC_50_ = 2.16 μM), with no cellular toxicity at the tested concentrations (≤10 μM). In a competitive in vitro binding assay against 415 human kinases (Eurofins), compound **20** tested at 1 μM concentration showed excellent selectivity, having only three kinases hit over 65%, with all of those also belonging to the AGC-family (ROCK2, PKA, and PKAcβ). To rationalize the binding mode, docking studies were performed with compound **20** based on a LATS1 kinase domain model obtained from the AlphaFold database. Based on the predicted LATS1/**20** complex, the high selectivity could be rationalized by Van-der-Waals and T-shaped stacking interactions between the LATS1 Phe1039 at the C-tail region and the central phenyl and pyrrolopyridine rings of inhibitor **20**. The same phenylalanine residue is conserved among the AGC kinase family members, which also explains the aforementioned off-targets. The pyrrolopyridine ring is also essential for the two canonical hydrogen bond interactions with the hinge region. Moreover, the model predicted the basic nitrogen of the piperidine tail to perform ionic interactions with the acidic residues Asp789 or Asp832 at the sugar pocket of the ATP site, which may play a role in the selectivity over the other AGC-family members.^87^

Experiments using wild-type and genetically modified cell lines confirmed that the induction of YAP signaling and cellular proliferation occurs via LATS inhibition by compound **20**. Due to the good oral bioavailability of **20**, together with its long half-life and the confirmed inhibition of YAP phosphorylation in mice, extensive in vitro experiments were performed to examine the behavior of inhibitor **20** in stem cells and organoids. In a three-dimensional (3D) human skin model, **20** demonstrated the role of the YAP pathway in the proliferation and differentiation of keratinocytes. Compound **20** was also very effective at promoting liver regeneration. After initial data showing that **20** affected biliary epithelial cell (BEC) organoids in driving liver regeneration, in vivo studies in murine models of partial hepatectomy (PHx) demonstrated a significant induction of hepatocyte proliferation in a dose-dependent manner after a single dose (30 or 100 mg/kg).^87^ In addition, extended hepatectomy (eHx) mouse models with removal of up to 86% of the liver showed increased liver weight and hepatocyte proliferation in all three lobular regions upon compound **20** treatment, which was accompanied by a pro-proliferative effect on the kidney. The effect of LATS inhibition by compound **20** was also evaluated in murine intestinal stem cells. Further studies in organoids and mice (5× 100 mg/kg, oral) showed increased proliferation and a reduction in the differentiation ability of several organs, thus limiting the translational potential for liver regeneration.^87^ Nevertheless, compound **20** is a powerful chemical tool to investigate further phenotypes and potential applications of LATS pathway inhibition.

## MAP2K4

The dual-specificity kinase MAP2K4 (Mitogen-Activated Protein Kinase Kinase 4), also known as MKK4, is a member of the stress-activated protein kinase (SAPK)/mitogen-activated protein kinase (MAPK) signaling pathway, and its activation is triggered by different cellular stress conditions.^88^ MKK4 phosphorylates the downstream kinases JNK1–3, which are also subject to phosphorylation by MKK7. Additionally, but to a lower extent, p38 MAPK isoforms also undergo activation by MKK4.^89,90^ In a beautiful work published in 2013, the Zender group identified MKK4 as a key molecular target to promote liver regeneration.^91^ Genetic approaches using short hairpin RNA (shRNA) in mouse models demonstrated that MKK4 silencing induces MKK7 and a JNK1-dependent activation of the transcription factors ATF2 and ELK1, which leads to an increased regeneration of hepatocytes.^91^ Despite the genetic data showing the potential to explore MKK4 as a target against liver diseases, the literature was limited to weak or nonselective small-molecule inhibitors^92^ such as the dual MKK7/MKK4 inhibitors **21** ^93,94^ and **22**,^95^ and the inhibitors **23**, **24**, and **25** (Figure 5), which primarily target other kinases but feature micromolar off-target activity on several MKK isoforms.^96^ The dual MKK4/7 covalent inhibitor **26** ^97^ (Figure 5) and the 3-arylindazoles derivative **27** ^98^ (Figure 5) showed a slight preference for MKK4 over MKK7 but are not selective enough to study the specific function of MKK4.

**Figure 5:**
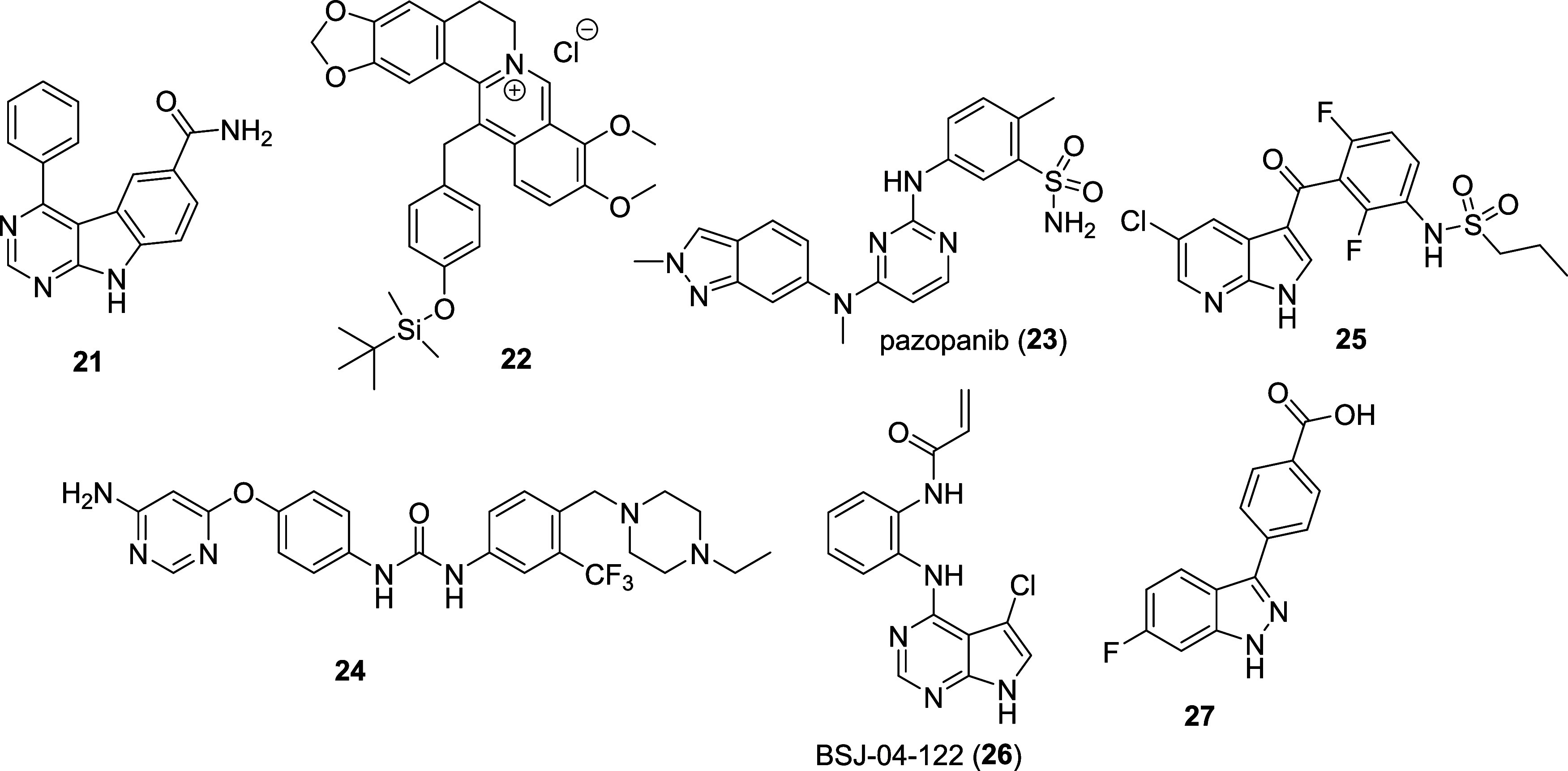
Molecular structure of the nonselective MKK4 inhibitors.

During the last years, the groups of Laufer and Zender have developed several potent and selective MKK4 inhibitors.^99–101^ The researchers started an academic drug discovery program, where the target hypothesis was validated by the generation of an in vivo probe and subsequent optimization into a clinical candidate. The FDA-approved BRAF^V600E^ inhibitor vemurafenib (**28**, Figure 6), which has MKK4 as one of its off-targets, served as the starting point, and optimization was supported by NMR structures since no high-quality X-ray data could be obtained. Vemurafenib binds to MKK4 in a nanomolar range (*K*_d_ = 13.5 nM) and inhibits the pMKK4-mediated phosphorylation of its downstream kinase JNK1 (IC_50_ = 0.8 μM).^102^ The main goal of the optimization was improving MKK4 potency while concomitantly increasing selectivity over Raf-members, as well as JNK1 and MKK7, which are crucial to the process of liver regeneration. In 2020, Laufer and Zender first published highly potent inhibitors such as compounds **29** and **30** (Figure 6), which had outstanding selectivity for MKK4 versus the off-targets MKK7, JNK1, MAP4K5, ZAK, and BRAF.^99^ Shifting the central scaffold from 1*H*-pyrrolo [2,3-*b*]­pyridine to 1*H*-pyrazolo­[2,3-*b*]­pyridine or a tricyclic α-carboline followed by an iterative SAR analysis yielded additional highly potent and selective MKK4 inhibitors, exemplified by compounds **31** ^100^ and **32** ^101^ (Figure 6). Follow-up work together with the spin-off Heparegenix has recently resulted in the development of darizmetinib/HRX-0215 (**33**, Figure 6), the first highly potent, selective, and orally available MKK4 inhibitor that has entered in clinical trials^102^ as well as its follow-up compound HRX-0233 (**34**, Figure 6).

**Figure 6:**
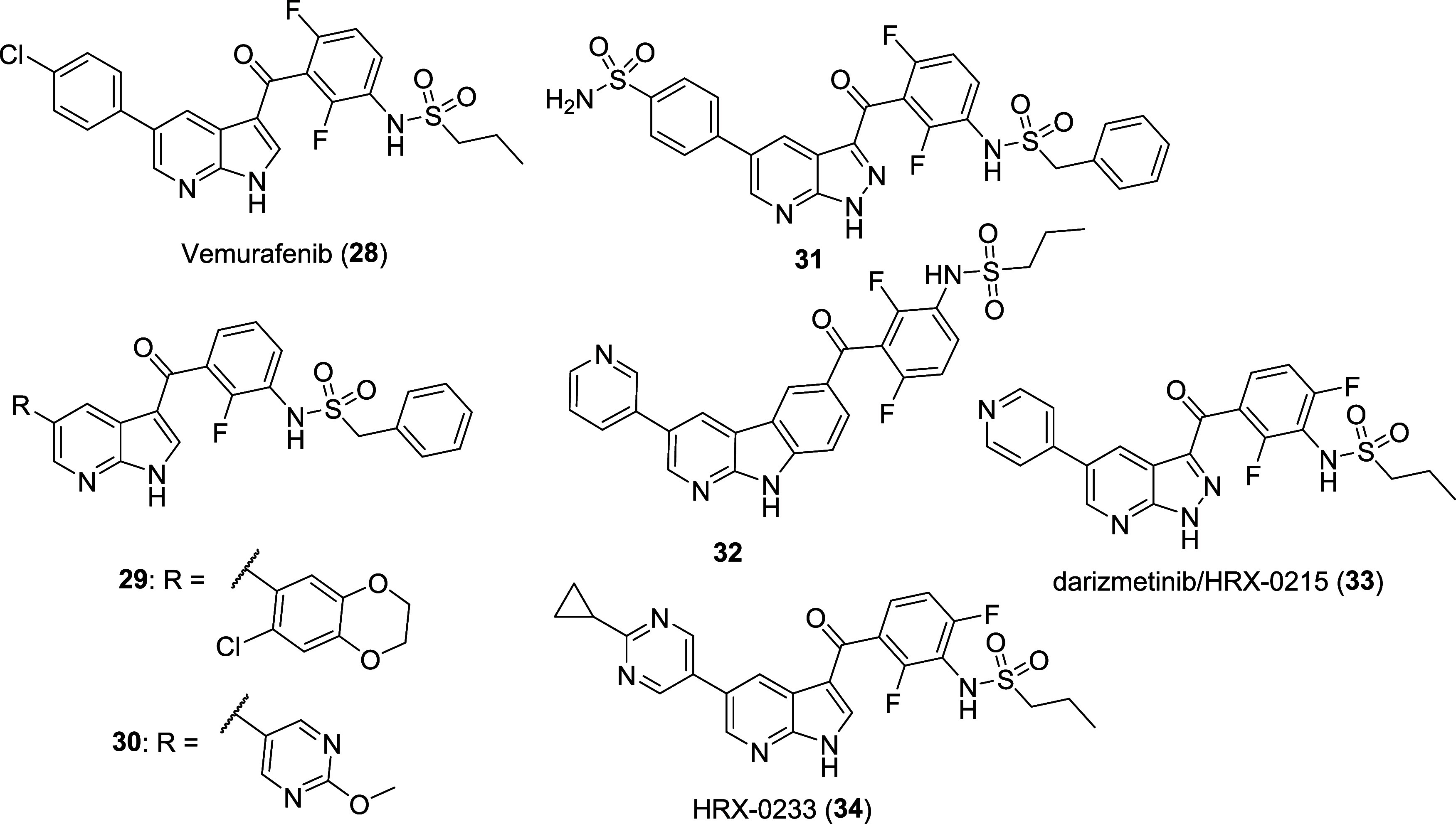
Molecular structure of the prototype compound vemurafenib (**28**), highly selective MKK4 inhibitors **31**, **29**, **30**, and **32** and chemical probe/clinical candidates darizmetinib/HRX-0215 (**33**) and HRX-0233 (**34**).

During the development of candidate **33**,^102^ an extensive SAR evaluation was performed at different parts of the structure. An initial ligand-based drug design (LBDD) approach revealed the carbonyl linker together with the alkyl sulfonyl group to be important features and a 1*H*-pyrazolo­[3,4*-b*]­pyridine system as the best hinge-binding moiety. The variation of the difluoro substitution pattern at the aryl linker was crucial to induce selectivity for MKK4. NMR experiments analyzing NOE correlations between mutated versions of the MKK4 kinase domain and some selected ligands gave a model that provided valuable insights into the binding mode supporting optimization. The key compound **33** showed nanomolar MKK4 inhibitory potency (IC_50_ = 20 nM) and a high degree of selectivity of more than 100-fold over the off-/antitarget kinases JNK1, BRAF-WT, and MKK7 (IC_50_ = 7.07, 11.46, and 14.97 μM, respectively). While selectivity was supported by comparative mRNA sequencing and transcriptome analyses of shRNA-mediated MKK4 knockdown vs **33**-treatment in hepatectomized livers, a broader kinome profiling of the compound remains to be disclosed in the literature. Phosphorylation of MKK4 was inhibited in a dose-dependent manner in cellular studies. Initial in vivo pharmacokinetic data in mice demonstrated a suitable elimination half-life (3.4–3.9 h) and superior maximum serum concentration (*C*_max_) after oral administration of 30 mg/kg compared to an equivalent dose of prototype **28**. Subsequently, an extensive amount of pharmacodynamic data was generated from mouse experiments, demonstrating the efficacy of candidate **33** in promoting hepatocyte proliferation and liver regeneration as well as a very good safety profile in rodents and nonrodents. Moreover, in a porcine hepatectomy model with a lethal liver resection of 85%, compound **33** promoted remarkable liver regeneration with an unaltered intracranial pressure value, the latter being associated with hepatic failure in humans. Candidate **33** completed phase 1 clinical trials in healthy volunteers, where it showed a favorable pharmacokinetic profile and tolerability^102^ and the compound recently progressed to phase 2a.

To date, compound **33** is the best chemical tool available for studying MKK4 biology in vitro and in vivo. Yet a suitable negative control compound as well as a profiling against a larger kinase panel have not been disclosed in the literature and would further improve the value of compound **33** as a chemical probe. Until disclosure of the overall selectivity profile, careful interpretation of the results obtained with this compound used as a chemical probe is recommended.

## CITK

The Citron Rho-interacting kinase (CITK or CIT) is a serine/threonine kinase member of the AGC superfamily being closely related with the ROCK and AURK proteins.^103,104^ Many in vivo and in vitro genetic studies of induced CITK knockdown through siRNA demonstrate a pivotal role of this protein in cytokinesis, suggesting CITK inhibition as a promising strategy for the treatment of different kinds of cancers, such as medulloblastoma, multiple myeloma, and prostate cancer.^105–110^ In addition, complementary genetic analysis using CRISPR also showed the relevance of CITK activity in the brain.^111^ Despite this accumulated data emphasizing the genetic link of CITK with diseases, the translatability of the CITK inhibition remained unexplored due to the lack of a high-quality chemical probe.^112^

Recently, Maw and co-workers^113^ developed the first potent and selective CITK inhibitors by repurposing reported kinase inhibitors, which have CITK as an off-target. After analyzing approximately 250 kinase inhibitors from previous studies,^114,115^ they confirmed 11 compounds as promising starting points (defined by a CITK IC_50_ < 1 μM). A preliminary optimization of the known ROCK inhibitor **35** (Figure 7A, CITK IC_50_ = 150 nM) by moving the amide group to the terminal portion of the molecule yielded the slightly less potent but chemically more stable **36** (Figure 7A, CITK IC_50_ = 250 nM). Extensive SAR exploration at the terminal portion, hinge-binding region, and the biaryl central core were performed in order to increase CITK affinity as well as selectivity. Besides the rather unusual methyl group at the C2 of the azaindole hinge-binding moiety (see Figure 7A, in red) and a slight potency improvement after introduction of methyl groups at the terminal alkyl portion (see Figure 7A, in blue), the addition of a key methyl (see Figure 7A, in green) positioned at the *ortho*-position on the azaindole side of the phenyl linker moiety boosted the selectivity of the compound C3TD879 (**37**, Figure 7A, CITK IC_50_ = 11 nM) over the main off-targets (ROCK1, JAK1, AKT1, AURKB, and IKK-β).^113^ To rationalize the methyl’s role in the selectivity, an X-ray cocrystal structure of the AGC-family member PKAα, which shares an almost fully conserved ATP-binding pocket with CITK, was obtained with analogue **38** (Figure 7A,B) (PDB code: 8SF8). Visual inspection of the hydrophobic surface area strongly suggests that the methyl groups of compound **37** lead to steric clashes at the off-targets and that CITK could accommodate bulkier groups better, possibly due to a higher flexibility at the “AGC-loop.” Moreover, the high selectivity of **37** toward CITK was confirmed in a panel of 373 human kinases (Eurofins), with only 5 other kinases having <50% residual activity at a concentration of 1 μM (AAK1, MNK2, ACK1, BIKE, and DRAK2). Follow-up biochemical dose–response experiments showed that only the closely related NAK-family members AAK1 and BIKE had an IC_50_ < 1 μM (212 and 902 nM, respectively), indicating a selectivity of >17- and 75-fold, respectively, for CITK.^113^

**Figure 7:**
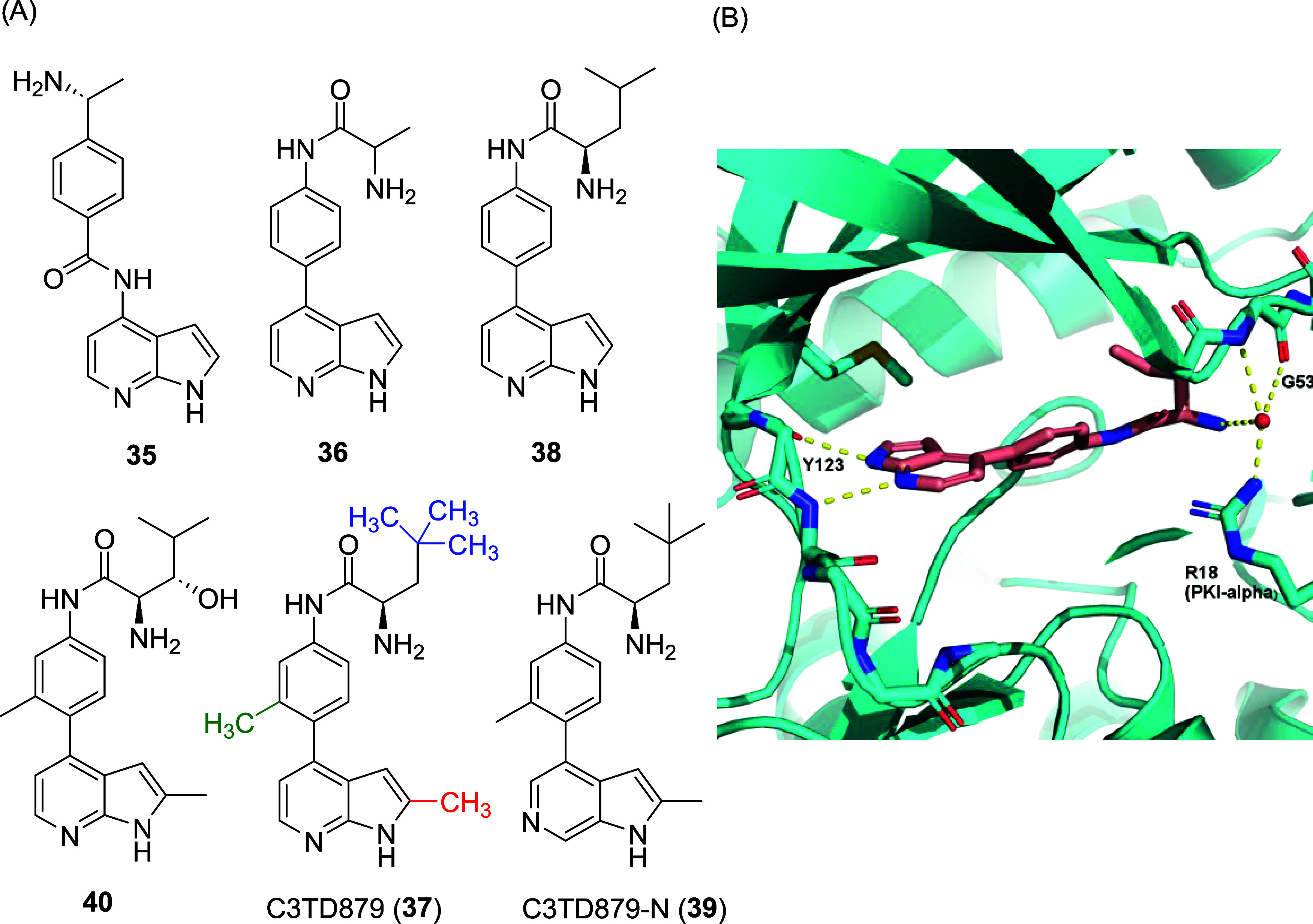
(A) Molecular structure of the hit CITK inhibitors **35**, **36**, optimized inhibitors **38**, **40**, and CITK chemical probe C3TD879 (**37**) with its negative control C3TD879-N (**39**). (B) Binding mode of **38** to the ATP-binding pocket of PKAα, used as a surrogate for the AGC-family kinases, and the Arg18 of a peptide (PKI-α) used in the crystallization procedure.

Interestingly, the researchers showed that inhibitor **37** induces a thermal shift of only ∼2 °C in differential scanning fluorimetry (DSF) experiments using the CITK kinase domain, although being used at a very high concentration of 100 μM. However, potent CITK engagement of **37** was confirmed in living HEK293 cells by NanoBRET assays (*K*_d_ = 0.3 nM or 9.5 nM using kinase domain or full-length kinase, respectively), with a consistent direct relationship between on-target and cellular NanoBRET activity. A promising profile of **37** was also demonstrated in an initial in vitro pharmacokinetic evaluation, including low microsomal clearance and CYP inhibition, and high permeability. Moreover, experiments in vivo highlighted an excellent bioavailability after oral administration (72% in rats) and high systemic exposure in rats and mice.^113^ Together, these results support the use of compound **37** as a high-quality chemical probe to shed light on the roles of CITK in health and disease. Notably, the compound was provided together with the negative control C3TD879-N (**39**, Figure 7A), where the hinge-binding pyridine nitrogen atom was shifted by one position.

These compounds were used in target validation studies, where initial experiments in HeLa cells did not demonstrate a specific antiproliferative effect after 48 or 164 h of cellular exposition to the probe **37** at 10 μM maximum concentration. In an NCI60 panel of 60 different tumor cell lines, **37** also had no effect below 1 μM. Corroborating the negative results, even the most potent CITK inhibitor **40** (Figure 7A, IC_50_ = 5.5 nM) did not demonstrate antiproliferative activity with an IC_50_ value better than 4 μM against approximately 900 cancer cell lines tested from the PRISM panel from the Broad Institute. Moreover, no significant differences were observed in the induction of multinucleation or changes in the distribution of the cells in the cell cycle between **37**, its negative control **39**, and DMSO in cytokinesis experiments.^113^ These data show that inhibition of CITK kinase activity does not correlate with the antiproliferative phenotype observed in genetic models of CITK loss, suggesting that structural features of CITK other than its catalytic function may play a more important role in cancer cell growth.

## Summary and Conclusions

Establishing a robust genetic link between a molecular target and a disease considerably improves the chances of success of a drug candidate in clinical trials.^116^ Yet, complementary pharmacological approaches relying on chemical probes are key to successful target validation and translation.^24^ The highly interdisciplinary nature of drug discovery involves a wide range of scientists from different research fields, who may not always be familiar with the criteria of proper probe selection and application.^117^ Thus, well-characterized and openly accessible chemical tool compounds, together with comprehensive and easily accessible information on their properties and limitations, are essential for advancing basic and translational biomedical research. Given the enormous translational impact of protein kinases, the development of high-quality chemical probes to explore the function of the understudied and chemically unexplored fraction of the kinome is a highly relevant area of research.^30,31^

Here, we provide an overview of chemical probes for in vivo use published and deposited in the Chemical Probes Portal in 2024. We highlight compound Wee1-IN-7 (**3**)^63^ as the best available chemical probe for in vitro and in vivo studies of this kinase and describe advances in the development of subtype-selective chemical probes for SIK kinases such as **13** ^79^ and GLP3970 (**14**).^80^ The generation of the best-in-class chemical probe **20** ^87^ and the inhibitor darizmetinib/HRX-0215 (**33**)^102^ for the protein kinases LATS1/2 and MKK4, respectively, represents an important step not only to chemically complement the existing genetic studies for these proteins but also to validate these kinases as promising new drug targets, among others, for liver regeneration and treatment of liver failure. Moreover, and as nicely described in previous reports on the chemical probes BAY-985 ^118^ and SGC-CK2-1 ^119^ targeting the protein kinases TBK1 and CK2, respectively, the free availability of high-quality tool compounds is critical to counteract scientific misinformation and provide reliable links between biological phenotypes and target proteins. Along the same lines, in vitro and in vivo studies with the high-quality chemical probe C3TD879 (**37**)^113^ demonstrated that selective inhibition of the CITK kinase function does not reproduce the antiproliferative cellular phenotype observed for genetic CITK loss. While this may limit CITK inhibition as a strategy in cancer treatment, the availability of a chemical tool now enables the exploration of CITK inhibition in other diseases. Notably, the availability of additional degrader probes would be very helpful to assess whether chemical knockdown can reproduce the genetic phenotype in cases where it does not seem to be linked to catalytic function.

An overview of all probes discussed in this Review is shown in Table 1.

**Table 1:** Summary of the High-Quality Chemical Probes Discussed in this Review^a^

kinase target (s)	chemical probe	negative control	recommended concentration for cellular use	link to the chemical probe portal	primary literature ref
Wee1	Wee1-IN-7 (3)	n.r.	up to 1 μM	ref 120	wang et al.^63^
SIK2/3	GPLG3970 (14)	n.r.	up to 1 μM	ref 121	peixoto et al.^80^
SIK1/2	JRD-SIK1/2i-4 (13)	n.r.	5 to 10 μM	ref 122	babbe et al.^79^
LATS1/2	NIBR-LTSi (29)	n.r.	up to 5 μM	ref 123	namoto et al.^87^
MAP2K4	HRX-0215 (33)	n.r.	up to 3 μM	ref 124	zwirner et al.^102^
CITK	C3TD879 (37)	C3TD879-N (39)	up to 1 μM	ref 125	maw et al.^113^

a Data from the respective Chemical Probes Portal link. n.r.: not reported.

Unfortunately, unsuitable historic compounds (see a list from the Chemical Probes Portal)^126^ still remain in frequent use, thereby perpetuating erroneous interpretations of observed phenotypes and contaminating the scientific literature.^127^ In addition, a recent systematic literature review^128^ revealed that biomedical researchers are still far from implementing the best practices in the use of high-quality chemical probes, which includes their use in a wrong concentration or without negative control. We would like to (re)­emphasize that the promotion of the proper use of probes and tool compounds is similarly important as their development. Beyond following the previously discussed criteria, this also includes the use of at least two orthogonal probes, ideally from entirely different chemotypes in combination with suitable negative controls, whenever it is possible.

While this review exclusively focuses on chemical probes for in vivo use, it is worth mentioning that there are some additional well-characterized small-molecule kinase inhibitors deposited in the Chemical Probes Portal in the year 2024 that have been assessed solely in vitro. These compounds, including FGFR4-IN-8B^129^ (an FGFR4 inhibitor) and RLY-2608 ^130^ (a PI3Kα inhibitor), may serve as suitable tools for cellular studies. As mentioned before, new modalities such as heterobifunctional degraders, molecular glues, (ir)­reversible covalent inhibitors, and biological probes are now expanding and complementing the toolkit for chemical target validation by different mechanisms of action.^37,38^ Moreover, ambitious public-private alliances exemplified by Target 2035 ^131,132^ encourage researchers around the world to collaborate and join forces to develop novel chemical and biological tools as well as cutting-edge technologies to illuminate the entire proteome. The CACHE challenges^133^ and the Target 2035 podcast series^134^ represent noteworthy formats for the dissemination of relevant information and engagement of the broader scientific community, hopefully providing a source of inspiration for more such endeavors in the future.

Continued strong efforts for the development of high-quality chemical probes for protein kinases and beyond are needed to help academic laboratories and pharmaceutical companies to identify and validate molecular targets with an enhanced translational potential, improving the chances of achieving benefits to patients, especially for diseases with unknown molecular pathophysiology, in case of drug resistance, or where effective treatment options are completely absent.

## Abbreviations Used

AAK1: adapter protein 2-associated Kinase 1
ABL: abelson kinase
ACK1: activated Cdc42-associated kinase
AKT1: Ak strain transforming 1
AMPK: adenosine monophosphate-activated kinase
ATF2: activating transcription factor-2
ATP: adenosine triphosphate
AURK: aurora kinase
BEC: biliary epithelial cell
BMP2K/BIKE: BMP-2-inducible kinase
BRAF: V-Raf murine sarcoma viral oncogene homologue B
CaMK: calcium/calmodulin-dependent kinase
CDK: cyclin-dependent kinase
CITK: citron Rho-interacting Kinase
DRAK2: death-associated protein kinase-related apoptosis-inducing protein kinase 2
DSF: differential scanning fluorimetry
EGFR: epidermal growth factor receptor
eHx: extended hepatectomy
FDA: US Food and Drug Administration
GAK: cyclin-G associated kinase
HTS: high-throughput screening
IDH: illuminating the druggable genome
IKK-β: inhibitory kappa B kinase β
JAK1: Janus kinase 1
JNK1–3: Jun N-terminal kinase 1–3
LATS1/2: large tumor suppressor kinase 1/2
LBDD: ligand-based drug design
LCK: lymphocyte-specific protein tyrosine kinase
MAP2K4/MKK4: mitogen-activated protein kinase kinase
MAP3K19: mitogen-activated protein kinase kinase kinase 19
MAPK: mitogen-activated protein kinase
MKK7: mitogen-activated protein kinase kinase 7
MKNK2: MAP kinase-interacting serine/threonine-protein kinase 2
MSK1: mitogen and stress-activated protein kinase-1
NAK: numb-associated kinase
NIH: US National Institute of Health
NMR: nuclear magnetic resonance
NOE: nuclear overhauser effect
p70S6K: ribosomal protein S6 kinase β-1
PDB: Protein Data Bank
PHx: partial hepatectomy
PK/PD: pharmacokinetic/pharmacodynamic
PKAC: cAMP-dependent protein kinase
PLK: polo-like kinase
RIPK2: receptor-interacting serine/threonine-protein kinase 2
ROCK: Rho associated coiled-coil containing protein kinase
SAPK: stress-activated protein kinase
SAR: structure–activity relationship
SGK2/3: serum and glucocorticoid inducible protein kinase 2/3
SIK1–3: salt-inducible kinases 1–3
TECs: tubular epithelial cells
TNF-α: tumor necrosis factor α
YAP: yes-associated protein
